# Topological Parametric Amplification of Structured Interface States

**DOI:** 10.1002/nap2.70355

**Published:** 2026-11-10

**Authors:** Weijie Liu, Hongwei Gao, Xuchang Wang, Yao Wang, George F. R. Chen, Doris K. T. Ng, Dawn T. H. Tan

**Affiliations:** ^1^ Photonics Devices and System Group Singapore University of Technology and Design Singapore; ^2^ Institute of Microelectronics Agency for Science Technology and Research (A*STAR) Singapore

**Keywords:** integrated optics, parametric amplification, topological system, trimer lattice, ultra‐silicon‐rich nitride

## Abstract

Topological photonics provides a robust framework for light manipulation, but integrating coherent parametric processes with spatially structured topological states remains difficult. Here, we demonstrate topological parametric amplification within structured interface states. By constructing a domain wall in a one‐dimensional trimer lattice, we create topologically protected interface states distributed across two waveguides. Due to this state distribution, light injected into a single waveguide exhibits continuous spatial beating during propagation, which inherently reduces the spatial overlap among the interacting fields during parametric amplification. We theoretically analyze this impact on parametric amplification and experimentally utilize a platform with high third‐order Kerr nonlinearity to realize the parametric process. We achieve a signal gain of 3.5 dB and an idler conversion efficiency of −4.4 dB. These results show that parametric amplification can be sustained within structured topological interface states, providing a pathway to integrate coherent parametric processes into complex topological systems.

## Graphical abstract

Topological interface states in a trimer waveguide lattice support parametric amplification while light continuously exchanges energy between two interface waveguides. Despite the reduced spatial overlap of the interacting fields, an ultra‐silicon‐rich nitride (USRN) platform yields a signal gain of 3.5 dB. These findings extend topological parametric amplification to spatially distributed states with internal energy exchange.

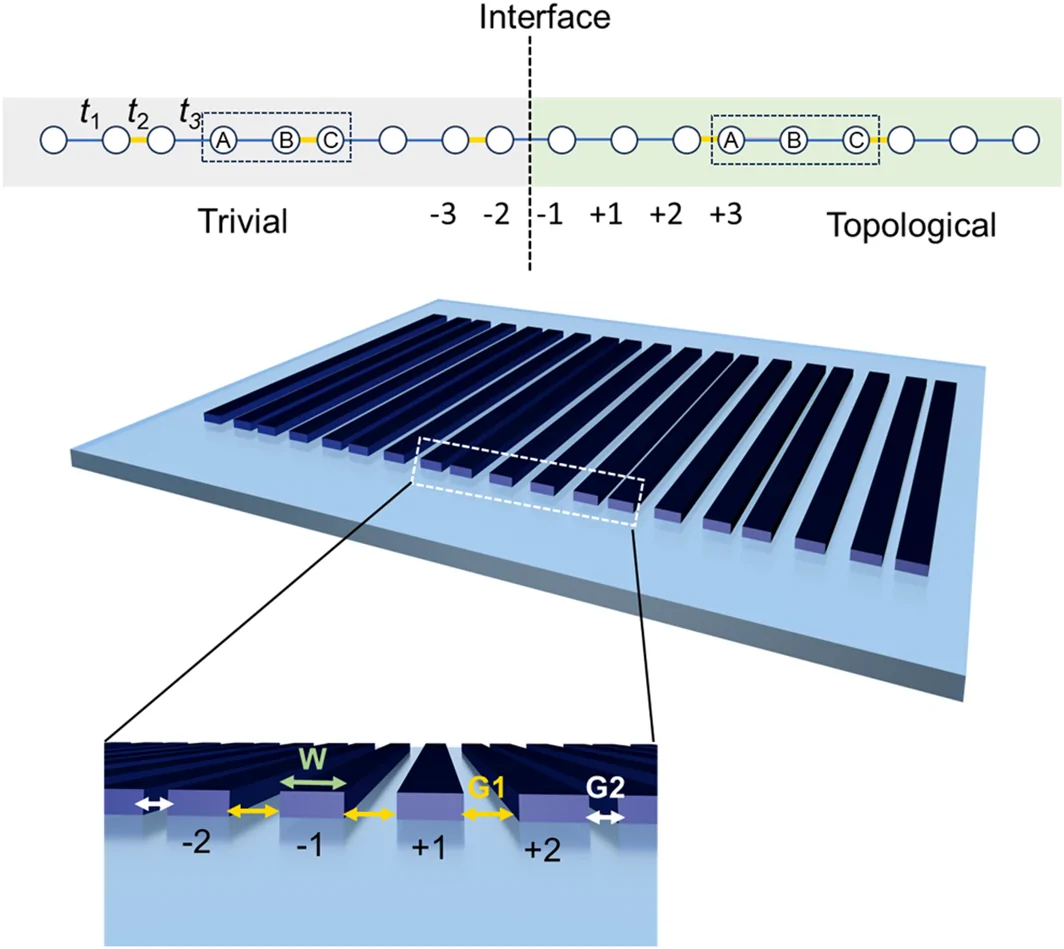

## 1 Introduction

The concept of band topology provides a physical mechanism to protect wave propagation against scattering and environmental disorder [1, 2]. The implementation of these concepts in photonic systems has established the field of topological photonics [3, 4]. This field has been further integrated with diverse physical frameworks, such as non‐Hermitian physics, Floquet systems and synthetic dimensions, to broaden the scope of light manipulation [5, 6, 7, 8, 9, 10, 11, 12, 13, 14, 15, 16, 17, 18]. Building upon these developments, the interplay between topological systems and optical nonlinearities has emerged as an active research direction [19, 20, 21, 22, 23]. In waveguide array system, the majority of experimental and theoretical efforts in nonlinear topological photonics have concentrated on intensity‐dependent phenomena [23, 24]. In these systems, local refractive index changes induced by the optical Kerr effect typically act as a perturbation to the topological structures or facilitate the formation of topological solitons [21, 25, 26, 27].

In contrast to these intensity‐dependent effects, the integration of coherent parametric processes, such as four‐wave mixing (FWM), presents distinct physical challenges. Efficient parametric generation requires high spatial overlap between the interacting fields [28, 29, 30, 31]. Integrated photonic platforms, particularly silicon and CMOS‐compatible photonics, provide an effective experimental system [32, 33, 34, 35, 36, 37, 38, 39, 40]. Benefiting from tight mode confinement and the strong effective nonlinearities available in specific materials, these platforms are suitable for investigating nonlinear topological phenomena. However, research on coherent parametric processes within these topological platforms remains limited, and existing experimental demonstrations have relied primarily on highly localized, single‐site topological states in the Su‐Schrieffer‐Heeger (SSH) lattice [41, 42]. In these SSH systems, strong confinement to a single waveguide provides a high spatial overlap of the interacting fields, with limited energy exchange between waveguides during the parametric process. Topological states can also extend over several lattice sites, allowing inter‐waveguide coupling to participate in the nonlinear interaction. Studying parametric amplification in such states provides insight into how the spatial evolution of the pump, signal, and idler affects their interaction. Differences in their spatial evolution reduce the spatial overlap integral among the interacting fields and consequently the nonlinear conversion efficiency compared to an isolated waveguide. However, investigating nonlinear interactions within these structured states remains essential, as their dynamic field evolutions naturally form the basis of more complex topological systems, extending to adiabatic evolution, Floquet models, and non‐Hermitian platforms.

In this work, we demonstrate the parametric amplification within structured interface states. Using a one‐dimensional trimer lattice, which is a variant of the SSH model, we create localized topological interface states distributed across two waveguides. Excitation of these interface states allows light to exchange energy between the two waveguides while remaining localized at the domain wall. The trimer lattice therefore provides a simple system for studying the interplay between spatial evolution and parametric amplification. In particular, this system allows the effects of spatial beating and its wavelength dependence on the parametric process to be investigated. Theoretical analysis reveals that the continuous spatial beating of the propagating optical fields reduces the spatial overlap integral, limiting the maximum parametric efficiency to 1/16 of that in an isolated waveguide. On the other hand, the optical intensity remains confined within the waveguide cores as the topological interface state propagates, resulting in a constant total optical intensity within the two cores. This ensures maximum interaction of the optical field with the highly nonlinear core as opposed to the cladding which has low nonlinearity. To ensure the observation of parametric amplification, we utilize the ultra‐silicon‐rich nitride (USRN) platform, taking advantage of its high third‐order Kerr nonlinearity $\chi^{3}$ and negligible two‐photon absorption at telecommunication wavelengths. After demonstrating the topological localization and continuous spatial beating in linear experiments, we conduct parametric amplification experiments and observe a signal gain of 3.5 dB and an idler conversion efficiency of −4.4 dB. These results demonstrate that coherent parametric processes can be effectively sustained within structured topological interface states, establishing a framework for investigating nonlinear interactions in more complex topological systems.

## 2 Design of the Trimer Lattice With a Domain Wall

We use a one‐dimensional (1D) trimer lattice to realize the topological domain wall. As shown in Figure 1a, the trimer lattice is a variant of the SSH model comprising three sites (A, B, and C) per cell [43, 44, 45]. Under the tight‐binding approximation, the Hamiltonian of a standard trimer lattice is expressed as:

(1) $$H_{0}=\sum_{n}\left(t_{1}a_{n}^{†}b_{n}+t_{2}b_{n}^{†}c_{n}+t_{3}c_{n}^{†}a_{n+1}+h.c.\right),$$

where $a_{n}^{†}\left(a_{n}\right)$ $b_{n}^{†}\left(b_{n}\right)$ and $c_{n}^{†}\left(c_{n}\right)$ represent the creation (annihilation) operators at sublattices A, B, and C within the *n*th cell, respectively. The hopping amplitudes (*t*_1_, *t*_2_, *t*_3_) determine the topological phase of the lattice. We establish a topologically trivial phase by setting the hopping sequence to (*t*_1_, *t*_2_, *t*_1_), and a topologically non‐trivial phase using the sequence (*t*_1_, *t*_1_, *t*_2_) [45]. Here, *t*_2_ and *t*_1_ refer to the strong and weak coupling coefficients (*t*_2_ > *t*_1_), respectively (more details in Supporting Information S1). To construct the complete system, the trivial domain on the left and the topological domain on the right are connected via the coupling *t*_1_. In the trimer waveguide array system, the spatial propagation of the optical fields along the longitudinal direction (*z*) is analogous to temporal evolution in quantum mechanics, governed by the Schrödinger‐like coupled‐mode equation: $id|\psi \left(z\right)\rangle /dz=H_{\text{total}}|\psi \left(z\right)\rangle$, where |ψ(z)〉 represents the state vector of the modal amplitudes across all waveguides at a given propagation distance *z*, and $H_{\text{total}}$ denotes the Hamiltonian of the waveguide array including the domain wall [46, 47].

**FIGURE 1:**
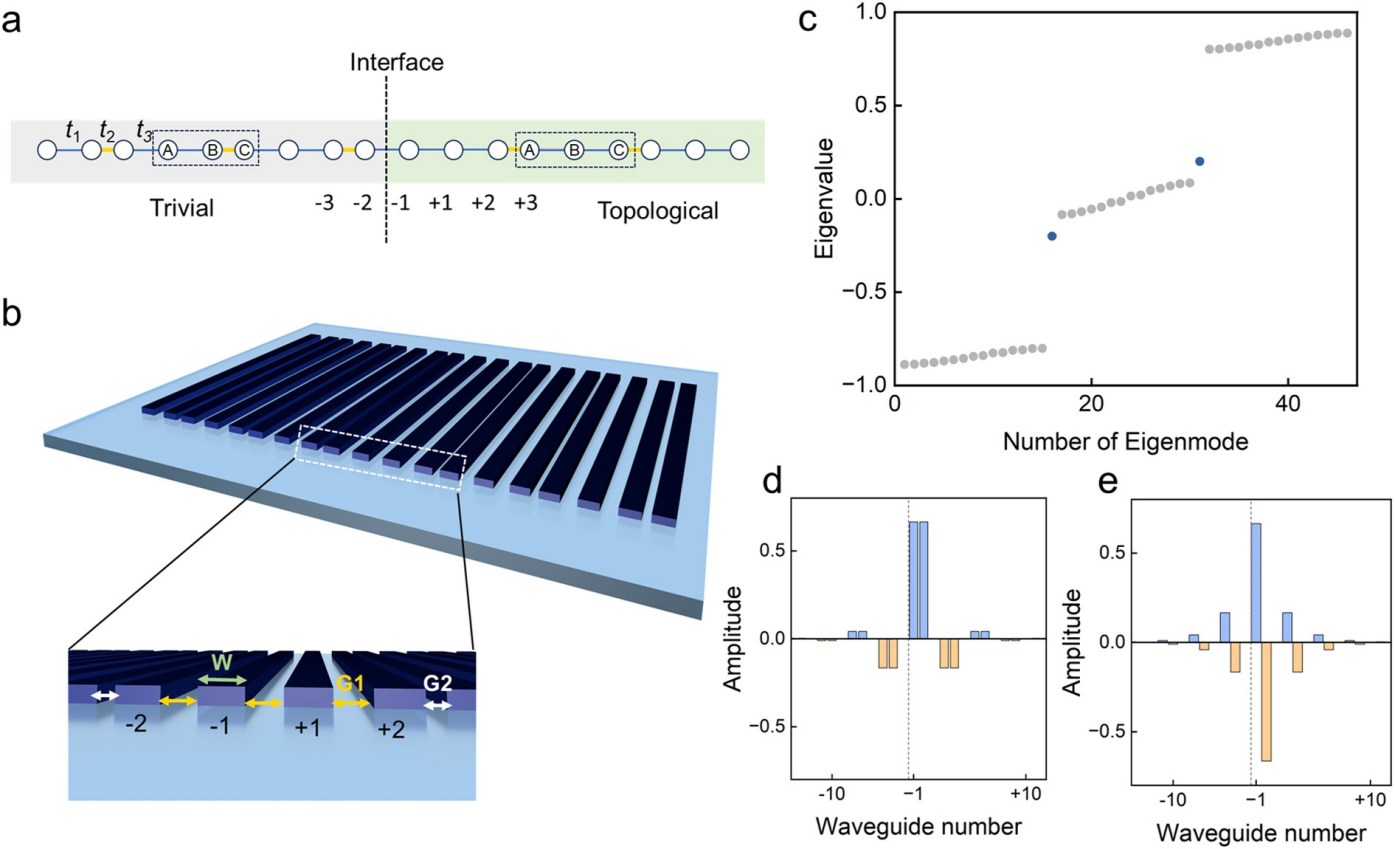
Trimer lattice with domain wall and the topological interface states. (a) Schematic of the 1D trimer lattice consisting of a trivial domain (left) and a topological domain (right). The unit cells are indicated by dashed rectangles. (b) schematic of the USRN waveguide array. The inset shows the cross‐section. (c) Calculated energy spectrum of the lattice. The blue dots indicate the two in‐gap topological interface states. (d, e) Calculated spatial amplitude distributions for the two eigenstates corresponding (c). The states are primarily distributed on the two waveguides (−1 and +1). USRN, ultra‐silicon‐rich nitride.

We fabricated the waveguide array on a CMOS‐compatible ultra‐silicon‐rich nitride (USRN) platform using electron‐beam lithography (Figure 1b). The USRN waveguides are embedded in SiO_2_ cladding and designed with a rectangular cross‐section of 600 nm in width and 300 nm in height (more details in Supporting Information S1). By setting the inter‐waveguide gap distances to *G*_1_ = 250 nm and *G*_2_ = 150 nm, we obtain the weak and strong coupling coefficients of *t*_1_ = 0.016 μm^−1^ and *t*_2_ = 0.093 μm^−1^, respectively. This design yields a ratio of *t*_1_/*t*_2_ = 0.17, which ensures a topological bandgap [42]. The complete array consists of 60 waveguides with a total propagation length of 4 mm. The USRN material provides a high third‐order nonlinear coefficient and avoids two‐photon absorption at 1550 nm, enabling efficient nonlinear parametric amplification. Using these coupling parameters, we calculate the eigenvalue spectrum of the trimer lattice (Figure 1c). The result reveals two distinct eigenvalues residing within the bandgap. Compared to the standard SSH model where topological modes are typically confined to a single lattice site, the more complex cell of the trimer lattice leads to a broader spatial distribution of its topological states. The amplitude distributions of these two states (Figure 1d,e) show that the optical fields are localized to the two waveguides (labeled −1 and +1), naturally forming the spatially structured states. These two modes correspond to the symmetric and anti‐symmetric supermodes of the waveguides characterized by distinct propagation constants. Consequently, injecting light into a single waveguide at the interface results in propagation characterized by periodic spatial beating, analogous to spatial Rabi oscillations. The coexistence of interface localization and inter‐waveguide energy exchange leads to a spatial overlap of the interacting fields that varies along the propagation direction during the parametric process.

## 3 Experimental Observation of Topological Localization

Next, we characterize the topological localization and spatial beating experimentally. Light is injected into one of the interface waveguides (labeled as the −1 site). First, we measure the output power across four relevant waveguides: the two waveguides where the topological states are localized (−1 and +1 sites), and their adjacent waveguides (−2 and +2 sites). As the wavelength of the injected light is swept from 1520 to 1600 nm, the measured transmission spectra for the trimer lattice (Figure 2a–d) reveal that the power at the interface waveguides (−1 and +1 sites) is much higher than that at the adjacent waveguides (−2 and +2 sites). This strong contrast verifies the topological localization of the interface states. The transmission spectra at the −1 and +1 sites exhibit rapid oscillations as a function of the input wavelength. Physically, the coupling coefficient between the two waveguides is wavelength‐dependent. Sweeping the input wavelength modifies this coupling coefficient, thereby changing the spatial period of the energy exchange. This variation leads to the periodic power oscillations observed at the 4‐mm output facet. An overlay of the two interface transmission spectra is provided in Supporting Information S1: Figure S4 of the Supporting Information, showing their out‐of‐phase oscillations with wavelength. The dashed lines in Figure 2 indicate the signal and pump wavelengths used in the FWM experiment presented in Figure 5.

**FIGURE 2:**
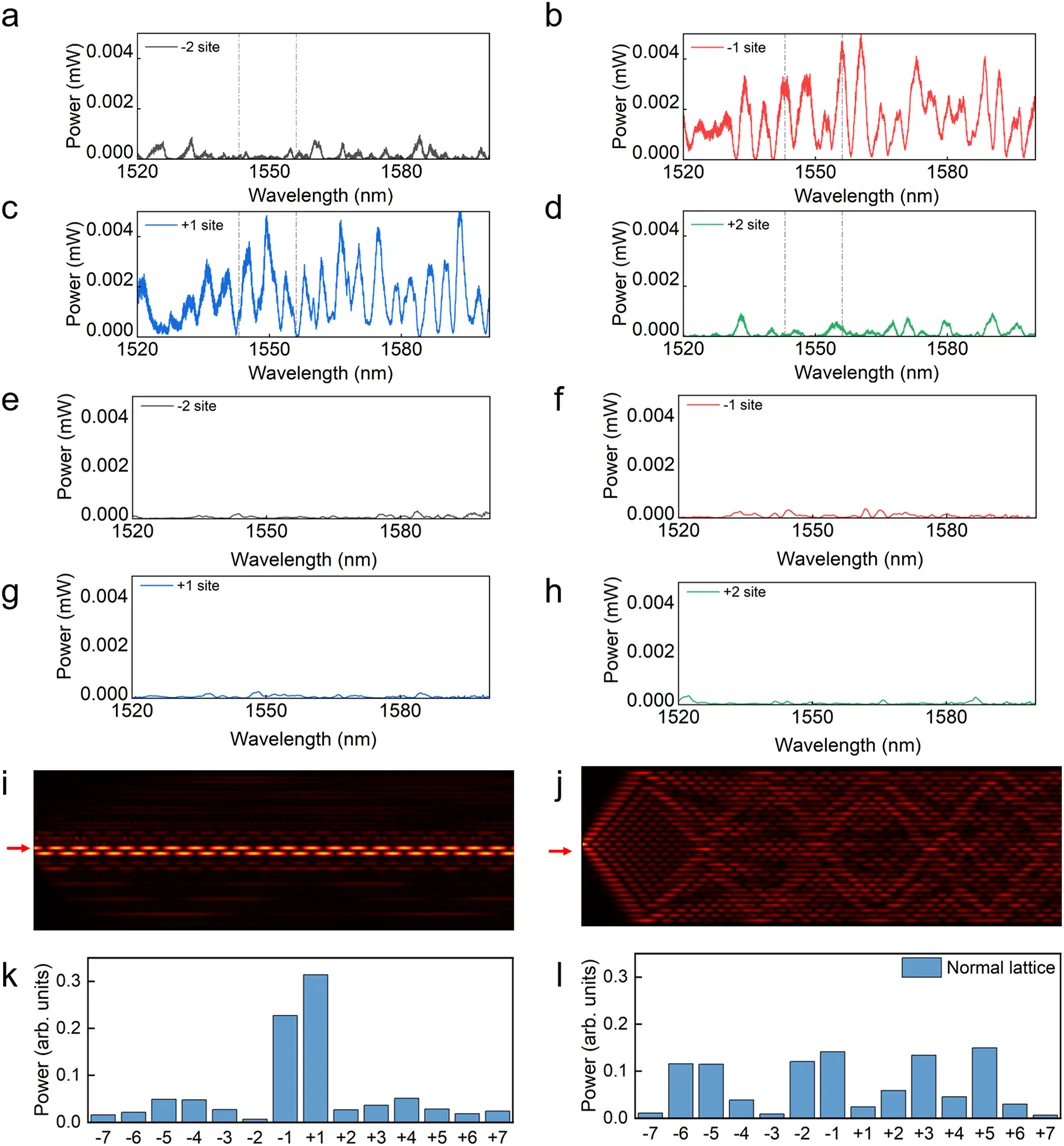
Linear experimental characterization and simulated spatial propagation dynamics. (a–d) Experimentally measured output transmission spectra of the interface waveguides and its adjacent waveguides for the trimer lattice, with light injected into the −1 site. (e–h) Corresponding output spectra for the trivial lattice under the same excitation. (i, j) Simulated spatial propagation profiles over a 4‐mm distance for the trimer lattice and the normal lattice, respectively. (k, l) Measured output power across 14 waveguides at a wavelength of 1550 nm for the trimer lattice (k) and the normal lattice (l).

For comparison, we perform identical spectral measurements on a trivial normal lattice, which consists of a uniform waveguide array with identical waveguide dimensions and a constant waveguide interval of G1. As shown in Figure 2e–h, the measured output powers at all four waveguides are low across the entire wavelength range. This indicates that the light diffracts into the bulk waveguides. We further experimentally verify this spatial confinement by measuring the output power distribution across 14 adjacent lattice sites at a fixed wavelength of 1550 nm. In the trimer lattice, optical power is predominantly concentrated at the −1 and +1 waveguides (Figure 2k). Under identical excitation, the normal lattice exhibits broad discrete diffraction across multiple waveguides (Figure 2l). Numerical simulations of the spatial propagation profiles for both structures are performed using the beam propagation method (BPM) in RSoft (Figure 2i,j). For the trimer lattice, the simulated optical field remains confined within the two interface waveguides, undergoing continuous, periodic spatial energy exchange. In the normal lattice, light injected into the excitation site rapidly diffracts and spreads deep into the bulk array.

## 4 Wavelength Dependence of the Interface States

Next, we examine in detail how wavelength variation affects the interface states. The simulation results show that $t_{1}$ and $t_{2}$ vary only slightly from 1540 to 1580 nm, as shown in Figure 3a,b. These variations have different effects on the interface localization and the spatial beating of the optical fields. Figure 3c shows the eigenvalue spectrum. Both interface‐state branches remain separated from the bulk bands, and the system stays in the same topological phase throughout this wavelength range. The interface‐state distributions at 1540 and 1580 nm are nearly identical (Figure 3d), indicating that the spatial distributions of the topological states remain almost unchanged over this wavelength range.

**FIGURE 3:**
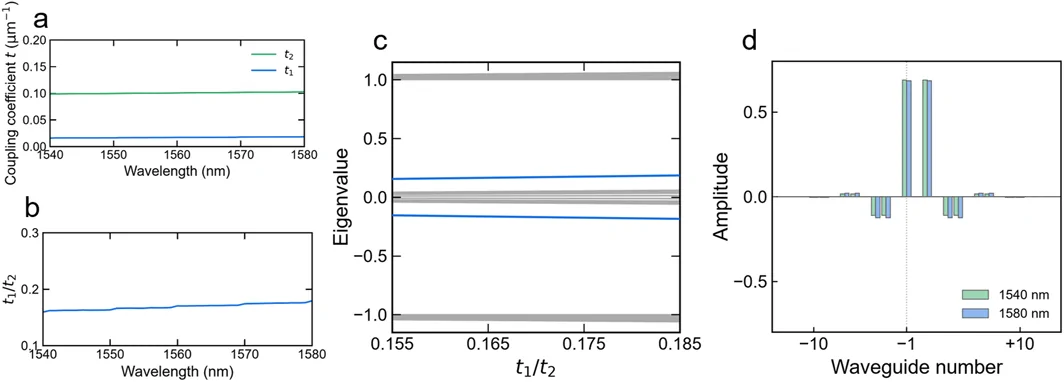
Wavelength dependence of the coupling and interface states. (a) Calculated coupling coefficients $t_{1}$ and $t_{2}$ as a function of wavelength. (b) Corresponding coupling ratio $t_{1}/t_{2}$ as a function of wavelength. (c) Eigenvalue spectrum as a function of $t_{1}/t_{2}$, with the interface states highlighted in blue. (d) Normalized amplitude distributions of the interface state at 1540 and 1580 nm.

The weak wavelength dependence of the coupling coefficients also produces only a small change in the spatial beating period. However, the 4‐mm propagation length spans many coupling lengths. Small differences in the beating periods therefore accumulate over propagation and can produce appreciable differences in the energy distribution between the two interface waveguides at different wavelengths. The interface localization is maintained, while the relative beating phases of the pump, signal, and idler can differ substantially over the interaction length. The following analysis neglects the small wavelength‐dependent differences in the interface‐state localization and retains the differences in the inter‐waveguide coupling dynamics. The pump, signal, and idler remain localized in the two interface waveguides, but exchange energy between them at different rates because their coupling coefficients differ. This approximation allows us to study how the different spatial evolutions of the pump, signal, and idler affect their spatial overlap and parametric amplification.

## 5 Theoretical Analysis of Parametric Amplification Within Structured States

We now analyze parametric amplification in the presence of these wavelength‐dependent spatial dynamics. In a degenerate FWM process, two pump photons ($\omega_{p}$) are annihilated to generate one signal photon ($\omega_{s}$) and one idler photon ($\omega_{i}$), governed by energy conservation: $2\omega_{p}=\omega_{s}+\omega_{i}$. In an isolated waveguide, efficient parametric generation requires a small phase mismatch, $\Delta \beta =2\beta_{p}-\beta_{s}-\beta_{i}$. In the trimer domain wall, the nonlinear interaction also depends on the spatial overlap of the fields as they exchange energy between the two interface waveguides. The effective coupling coefficients at the pump, signal, and idler wavelengths are denoted by $t_{p}$, $t_{s}$, and $t_{i}$, respectively.

Based on the previous theoretical and experimental results, light injected into one interface waveguide (e.g., the −1 site) undergoes continuous beating analogous to Rabi oscillations across the two waveguides. Therefore, the field evolution of the pump and signal at the −1 and +1 sites can be described by $\cos\left(t_{\lambda }z\right)$ and $i\;\sin\left(t_{\lambda }z\right)$, respectively. The nonlinear polarization driving the parametric generation is distributed across both waveguides. In the injected −1 site, the polarization is proportional to ${f_{-1}\left(z\right)=\cos}^{2}\left(t_{p}z\right)\cos\left(t_{s}z\right)$, whereas in the +1 site, it scales as $f_{+1}=-i\;\sin^{2}\left(t_{p}z\right)\sin\left(t_{s}z\right)$. The total idler field observed at the output facet of the −1 site is a coherent superposition of the idler generated in both waveguides, accounting for their subsequent propagation and cross‐coupling to the output facet. The effective idler amplitude accumulation is governed by the spatial overlap integral:

(2) $$A_{i}\left(L\right)\propto \int_{0}^{L}\left[\cos\left(t_{i}\left(L-z\right)\right)f_{-1}\left(z\right)+i\;\sin\left(t_{i}\left(L-z\right)\right)f_{+1}\left(z\right)\right]e^{i\Delta \beta z}dz.$$

This coupled spatial evolution results in two theoretical implications on the parametric efficiency compared to an isolated waveguide. The isolated‐waveguide reference also approximates the parametric process in an SSH domain wall when the interacting fields are strongly localized in a single interface waveguide. Its idler conversion efficiency is normalized to 1, allowing the effect of spatial beating in the trimer domain wall to be assessed relative to this strongly localized limit. Here, we assume that the system satisfies the phase‐matching condition. First, in an ideal scenario with perfectly matched coupling ($t_{p}=t_{s}=t_{i}$), evaluating this integral shows that the maximum attainable idler intensity is reduced to 9/16 of that in a single static waveguide (See Supplemental Material for details). Second, practical structure introduces a coupling mismatch. Assuming the coupling coefficients are equally spaced ($t_{p}-t_{s}=t_{i}-t_{p}$), the pump and signal fields oscillate with different spatial periods. As illustrated in Figure 4a, this difference causes them to walk off from each other along the propagation, further reducing the overlap integral. Figure 4b (blue curve) shows the calculated efficiency as a function of this coupling mismatch, defined as the difference in spatial oscillation cycles between the pump and signal fields over the interaction length. The theoretical maximum parametric efficiency is approximately 1/16 of the isolated waveguide case. To visualize this efficiency reduction, we calculate the idler intensity evolution along the propagation distance for both the two waveguide system and an isolated waveguide (Figure 4c). In this calculation, the coupling mismatch is set to 3. In the coupled system, the generated idler field undergoes continuous inter‐waveguide coupling, leading to strong intensity oscillations along the propagation. The envelope of this oscillating idler intensity is approximately 12 dB lower than the idler intensity in the isolated waveguide. This difference mathematically corresponds to the 1/16 efficiency ratio. We note that this calculation operates within the weak gain approximation, where the signal amplitude is assumed to be approximately constant. Under a strong gain regime, the exponential nature of parametric amplification would cause the coupled waveguide system to exhibit an even larger efficiency reduction relative to the isolated case. A detailed derivation of the spatial overlap integrals and the corresponding strong gain analysis are provided in the Supplementary Information. This theoretical limitation highlights the difficulty of observing parametric amplification in spatially coupled structures. It also motivates the use of the USRN platform: by utilizing a material with a high third‐order Kerr nonlinearity, we can offset this spatial overlap reduction and drive the parametric process into the experimentally observable regime. The trimer system poses limitations in the parametric amplification. However, this structural feature provides a distinct physical advantage. Rather than statically confining the nonlinear interaction to a single site, the continuous spatial beating couples the parametric energy conversion with the inter‐waveguide hopping dynamics. Furthermore, the parametrically generated idler field directly inherits the spatial localization and topological protection of the interface states.

**FIGURE 4:**
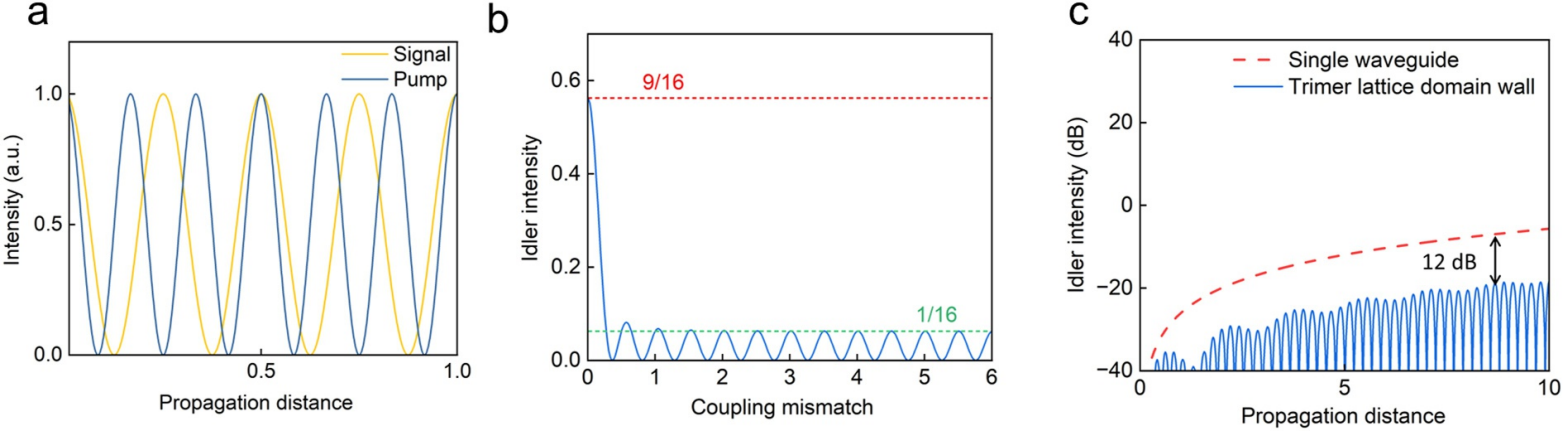
Theoretical analysis of parametric amplification efficiency in the trimer lattice. (a) Longitudinal spatial evolution of the pump and signal in the excited waveguide. The difference in their coupling coefficients ($t_{p}\neq t_{s}$) results in a spatial walk‐off. (b) Calculated normalized idler intensity generation as a function of the coupling mismatch. Compared to a static isolated waveguide (normalized to 1.0), the theoretical parametric efficiency is capped at a maximum of 9/16 under ideal coupling‐matched conditions, and drops to 1/16 under coupling mismatch. (c) Simulated idler intensity evolution along the propagation distance for an isolated single waveguide (red dashed line) and the coupled domain wall (blue solid line). The domain wall exhibits rapid oscillations and a 12 dB reduction in the idler envelope, corresponding to the 1/16 ratio shown in (b).

## 6 Experimental Observation of Topological Parametric Amplification

We experimentally investigate parametric amplification using the setup depicted in Figure 5a. A pulsed laser (∼5 ps pulse width, 20 MHz repetition rate) at *λ*_p_ = 1557.1 nm serves as the pump to provide sufficient peak power while reducing the refractive index increase induced by the Kerr effect. A continuous‐wave (CW) laser provides the signal at *λ*_s_ = 1544.1 nm. After being amplified and filtered, the CW signal is combined with the pump via a 3‐dB coupler and injected into the waveguide array. The output optical spectra are then recorded using an optical spectrum analyzer (OSA). Figure 5b displays the measured output transmission spectra as the input peak pump power is varied from 11.2 to 26.3 W. An idler peak emerges at approximately 1570.3 nm, satisfying the energy conservation relation of degenerate FWM. Simultaneously, sidebands appear around the signal wavelength. Such sidebands are also a characteristic feature of pulsed‐pump parametric amplification, as observed in SSH topological waveguides [42]. To assess the nonlinear performance under these spatially dynamic conditions, we calculate the signal gain and the idler conversion efficiency as a function of the input peak pump power (Figure 5c,d). As expected, both the signal gain and the idler conversion efficiency increase with higher pump power (more details in Supporting InformationS1). The system reaches a signal gain of 3.5 dB and an idler conversion efficiency of −4.4 dB at a peak pump power of 26.3 W. The generation of an idler, despite the theoretical spatial overlap reduction caused by the spatial beating, demonstrates the effectiveness of the nonlinear USRN platform.

**FIGURE 5:**
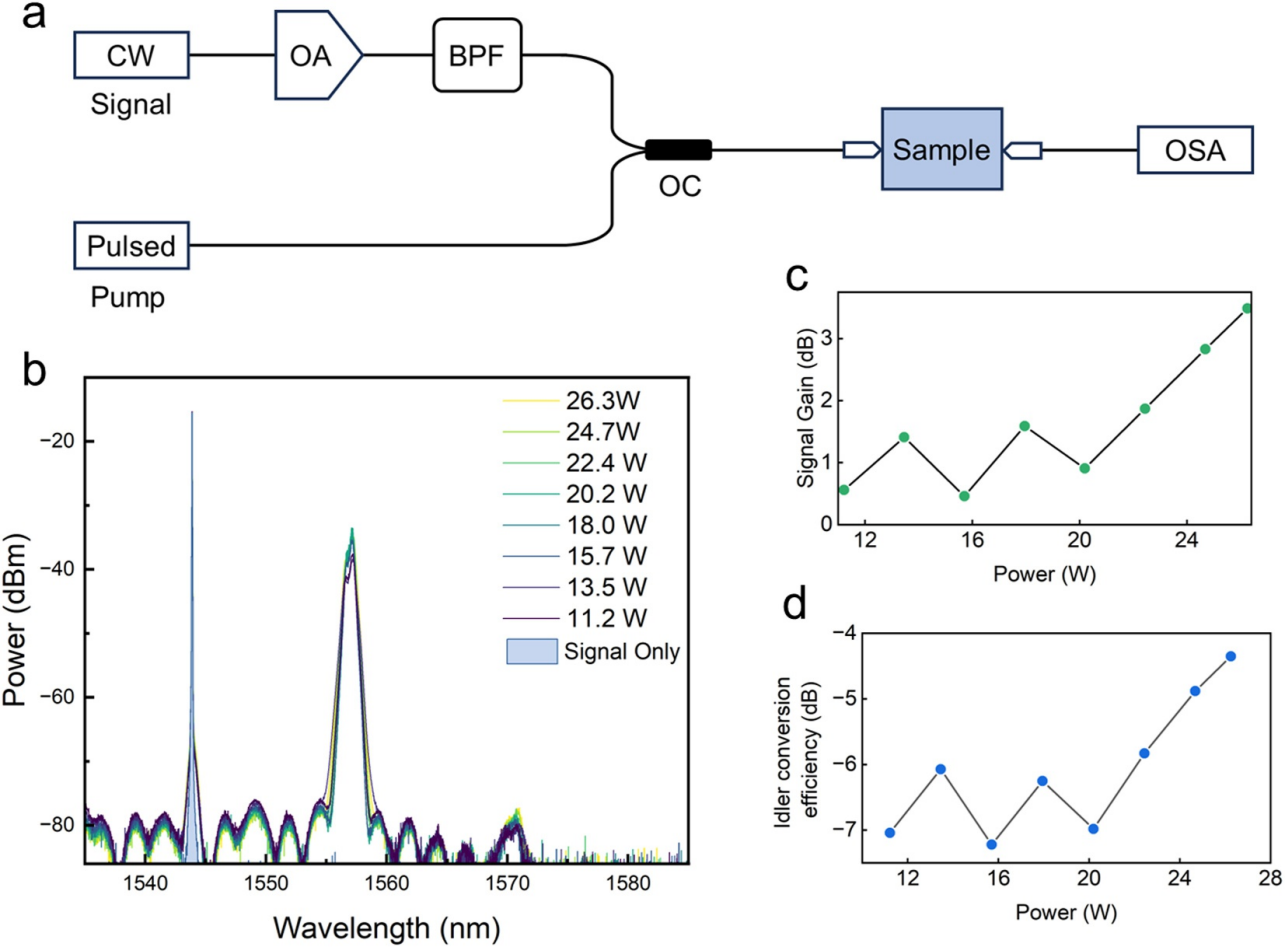
Experimental observation of parametric amplification as a function of pump power. (a) Schematic of the experimental setup for characterizing the nonlinear parametric process. CW, continuous‐wave laser; OA, optical amplifier; BPF, bandpass filter; OSA, optical spectrum analyzer. (b) Measured optical spectra at the output of the −1 site as the peak pump power is varied from 11.2 to 26.3 W, with pump and signal wavelengths fixed at 1557.1 and 1544.1 nm, respectively. An idler is generated at 1570.3 nm. (c, d) Measured signal gain (c) and idler conversion efficiency (d), both increasing with the input peak pump power.

We further investigate the wavelength tunability of the spatially dynamic parametric process. While maintaining a fixed peak pump power and fixed pump wavelength (*λ*_p_ = 1557.1 nm), the CW signal wavelength is discretely tuned in steps of 2.6 nm, specifically set to 1551.9, 1549.3, 1546.7, 1544.1, and 1541.5 nm. As shown in the spectra (Figure 6a), as the signal wavelength is tuned further away from the pump, the generated idler correspondingly shifts further away to longer wavelengths, governed by energy conservation. This demonstrates that the third‐order nonlinearity of the USRN platform sustains the parametric amplification across a broad spectrum. Figure 6b,c present the calculated signal gain and idler conversion efficiency at each signal wavelength. Tuning the interacting wavelengths simultaneously alters the phase‐matching condition and the spatial hopping amplitudes, continuously reshaping the effective overlap integral along the propagation.

**FIGURE 6:**
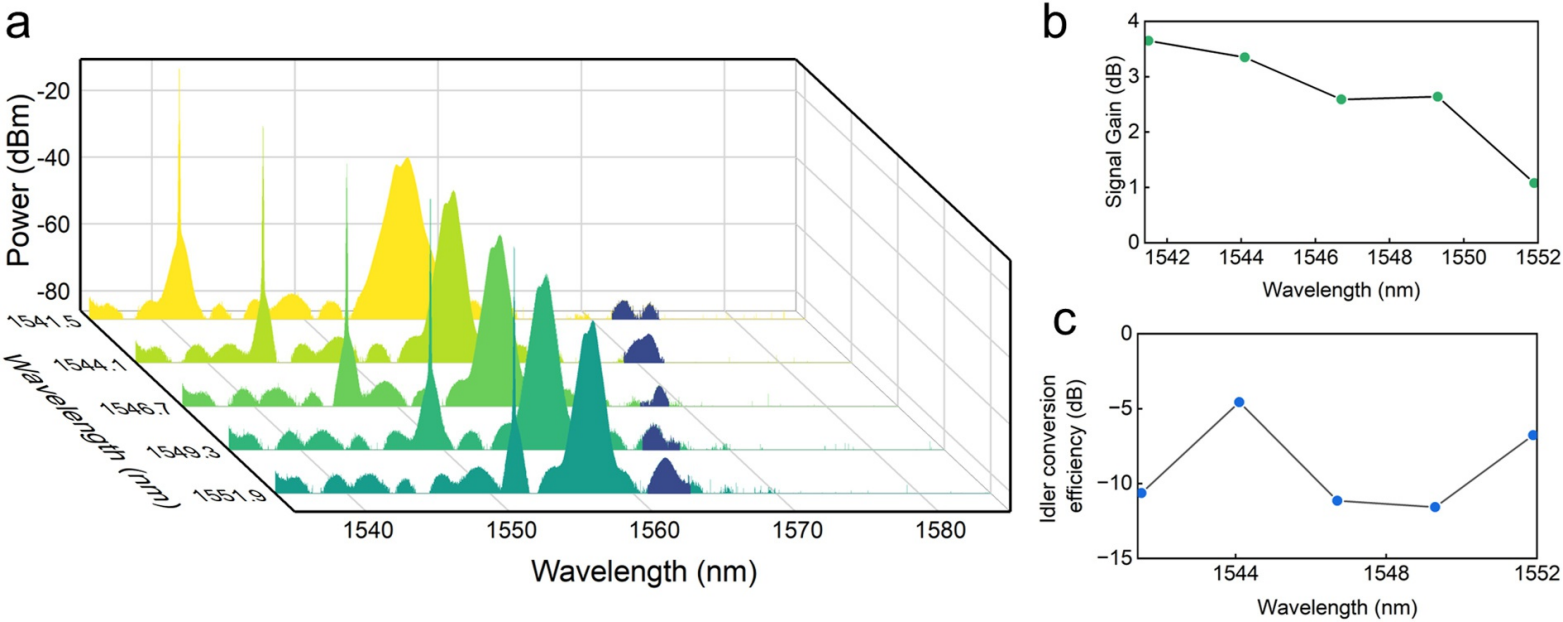
Wavelength tunability of the parametric amplification process. (a) Experimentally measured output spectra with the signal wavelength discretely tuned from 1551.9 to 1541.5 nm in steps of 2.6 nm (pump fixed at 1557.1 nm). (b, c) Measured signal gain and idler conversion efficiency at different signal wavelengths.

## 7 Topological Robustness of the Structured Interface States

Next, we investigate the topological robustness of the system. In our trimer lattice, the topological interface states originate from the non‐trivial domain on the right side of the interface, where the topological phase is strictly protected by spatial inversion symmetry. The left trivial domain lacks this symmetry. To verify the specific role of inversion symmetry, we introduce two distinct types of structural disorder to the coupling strengths. The perturbed coupling coefficients are defined as $t_{i}\left(1+\delta \right)$, where δ is a uniformly distributed random variable determining the disorder strength. For each disorder realization, the randomly perturbed coupling coefficients are fixed along the propagation direction. In the first disorder scheme (Figure 7a), the right domain is subjected to random perturbations on the inter‐cell couplings. This configuration preserves the inversion symmetry of the right domain, while random perturbations are applied to all coupling coefficients in the left domain. In the second scheme (Figure 7b), all coupling coefficients across the entire lattice are subjected to random perturbations, which completely breaks the inversion symmetry. We calculate the eigenvalue spectrum of the system as a function of the disorder strength for both cases (Figure 7c,d). When the system maintains inversion symmetry under the first disorder scheme, the eigenvalues of the topological modes exhibit minimal fluctuations even under strong disorder. Conversely, when the inversion symmetry is broken by the second disorder scheme, the eigenvalues fluctuate significantly and merge into the bulk bands. These spectral behaviors demonstrate that the topological interface states are strictly protected by the inversion symmetry. Consistent with these spectral findings, we simulate the spatial propagation of light under the first disorder type. As shown in Figure 7e, the optical fields remain strictly localized at the domain wall, and the localized light continues to exhibit periodic spatial energy exchange between the interface waveguides. The preservation of both the spatial confinement and the continuous spatial beating under symmetry‐preserving coupling disorder corroborates the topological robustness of the system.

**FIGURE 7:**
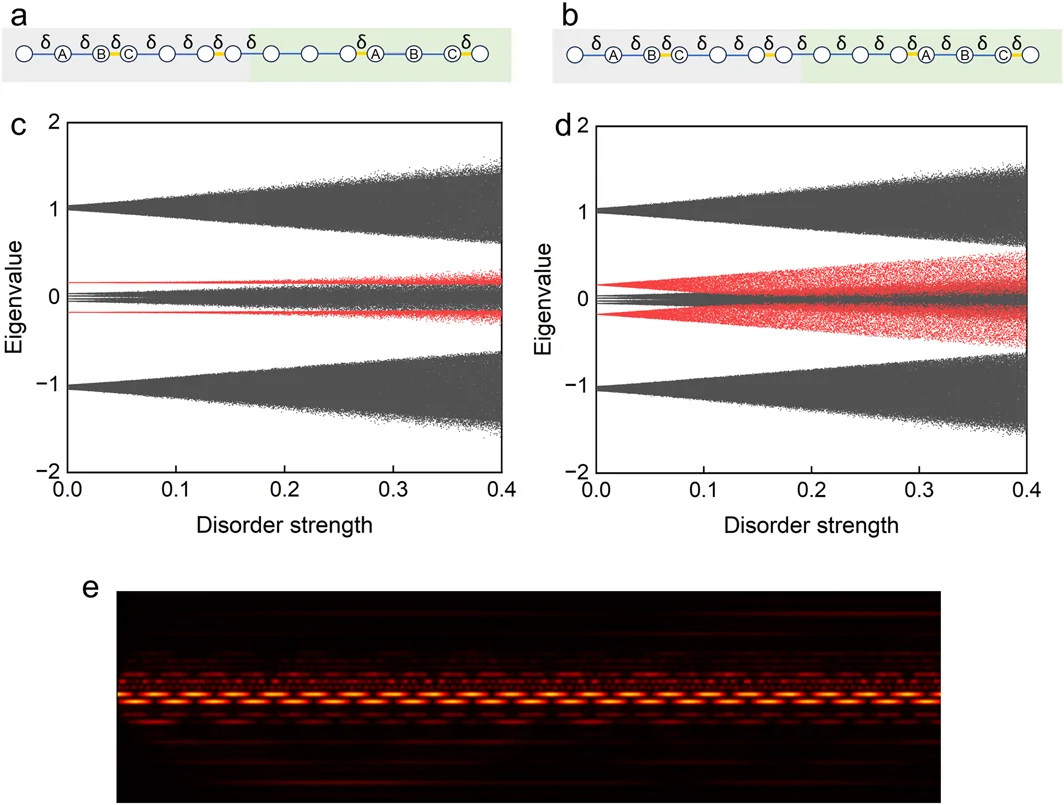
Topological robustness of the interface states in the trimer lattice. (a) Schematic of the trimer lattice under symmetry‐preserving disorder. Random perturbations are applied to the inter‐cell couplings in the right domain, while all couplings in the left domain are randomized. (b) Schematic of the trimer lattice under symmetry‐breaking disorder, where all coupling coefficients across the entire lattice are randomly perturbed. (c, d) Eigenvalue spectra as a function of the disorder strength corresponding to the disorder type in (a) and (b), respectively. The red markers highlight the eigenvalues of the topological states. (e) Simulated propagation profile under the first disorder type.

## 8 Discussion

We have experimentally demonstrated topological parametric amplification within spatially structured interface states. The domain wall in the 1D trimer lattice spatially confines the interacting optical fields, supporting topologically protected interface states distributed across two waveguides. This distributed profile causes the optical fields to undergo continuous spatial beating during propagation. Such spatial dynamics reduce the spatial overlap integral, inherently limiting the parametric conversion efficiency. By utilizing the high third‐order Kerr nonlinearity of the USRN platform, we effectively compensated for this reduced spatial overlap, achieving a signal gain of ∼3.5 dB and broadband idler generation.

Unlike an isolated pair of coupled waveguides, our system supports topological interface localization within an extended lattice. Controlling this localization through symmetry and the interface‐state positions relative to the bulk bands offers a way to manipulate parametric interactions and explore their extension to topological transport.

The significance of realizing topological parametric amplification within structured interface states extends beyond topological systems. First, our theoretical framework for idler efficiency provides general physical insights for FWM in complex waveguide arrays. This analytical approach can be readily applied to investigate nonlinear optical phenomena in other dynamic physical models, such as spatial Floquet topological systems and adiabatic evolution processes. Second, introducing a spatial degree of freedom into parametric processes holds potential for quantum information. Spontaneous FWM serves as a primary mechanism for generating entangled photon pairs [48]. Embedding this process within a structured interface states provides a robust method for generating path‐entangled biphoton states. The spatial beating inherently distributes the generated photons across discrete lattice sites, enabling spatially multiplexed quantum sources.

Furthermore, this work expands the scope of non‐Hermitian topological photonics. Current nonlinear studies in waveguide arrays primarily rely on intensity‐dependent Kerr effects, where local refractive index changes correspond to on‐site potential shifts. Parametric amplification, however, drives energy exchange among different wavelengths. Although the total energy of the interacting fields is strictly conserved, the single signal or idler field is effectively open. From the perspective of this single frequency, the energy exchange acts as an active gain, naturally manifesting as a non‐Hermitian process. Integrating active parametric gain with spatial light evolution provides a highly tunable platform for exploring non‐Hermitian topological phases.

## Author Contributions

**Weijie Liu:** conceptualization, methodology, software, data curation, investigation, formal analysis, validation, writing – original draft, visualization. **Hongwei Gao:** methodology. **Xuchang Wang:** methodology. **Yao Wang:** methodology. **George F. R. Chen:** methodology, investigation. **Doris K. T. Ng:** methodology. **Dawn T. H. Tan:** writing – review and editing, funding acquisition, project administration, conceptualization, resources, supervision.

## Supporting information


Supporting Information S1

## Data Availability

Data that support the plots within this paper and other findings of this study are available from the corresponding authors upon reasonable request.
