# Supplementary material for: Topological Parametric Amplification of Structured Interface States
Source: Nanophotonics. 2026 Nov 10;15(21):e70355. doi: 10.1002/nap2.70355 (PMC13749150; doi:10.1002/nap2.70355)
Supplement: Supplementary file 1 [file NAP2-15-e70355-s001.docx]

Supporting Information

Title Topological Parametric Amplification of Structured Interface States

Weijie Liu, Hongwei Gao, Xuchang Wang, Yao Wang, George F.R. Chen, Doris K. T. Ng and Dawn T.H. Tan*

**S1. Theoretical analysis of trimer lattice**

In this section, we introduce the physical properties of a one-dimensional trimer lattice. A trimer lattice consisting of three sites, A, B, and C, is considered, as illustrated in Figure S1a. Under the tight-binding approximation, the Hamiltonian of a trimer lattice containing *N* unit cells can be expressed as:

$$\begin{aligned} H_{0}=\sum_{n} \left( t_{1}a_{n}^{\dagger}b_{n}+t_{2}b_{n}^{\dagger}c_{n}+t_{3}c_{n}^{\dagger}a_{n+1}+h.c. \right)\#(S1) \end{aligned}$$

$t_{1}$ and $t_{2}$ are defined as the intra-cell coupling coefficients, while $t_{3}$ is the inter-cell coupling coefficient. By applying a Fourier transform, the Bloch Hamiltonian of the system can be written as:

$$\begin{aligned} H\left( k \right)=\left( \begin{matrix} 0 & t_{1} & t_{3}e^{-ik} \\ t_{1} & 0 & t_{2} \\ t_{3}e^{ik} & t_{2} & 0 \end{matrix} \right)\#\left( S2 \right) \end{aligned}$$

When the intra-cell couplings satisfy $t_{1}=t_{2}$, the system possesses inversion symmetry. This symmetry protects the topological phase, allowing its topological invariant to be characterized by the Zak phase. The Zak phase is defined as $\theta=\int_{-\pi}^{\pi} i\left\langle u\left( k \right)\left| \partial_{k} \right|u\left( k \right) \right.\rangle dk$, where $|\left. u(k) \right\rangle$ is the eigenstate of the Bloch Hamiltonian $H\left( k \right)$. Protected by inversion symmetry, the value of the Zak phase is strictly quantized to 0 or $\pi$, corresponding to the trivial and topological phases.^[1]^ Figure S1b shows the phase transition process of the system eigenvalues evolving with the inter-cell coupling $t_{3}$ when $t_{1}=t_{2}=1$. We consider a lattice consisting of 30 unit cells. When the parameters evolve to $t_{3}=1$, the energy gap closes. As $t_{3}$ continues to increase, the energy gap reopens, and the system enters the topological phase. Figure S1c provides the energy spectrum of this system with a parameter ratio set to $t_{3}/t_{1}=5$. Degenerate topological eigenstates clearly emerge within the bulk energy band gap. The amplitude distribution of a topological eigenstate is shown in Figure S1d, characterizing a symmetric localization at both the left and right ends of the one-dimensional lattice.

**Figure S1.** Topological properties of the trimer lattice. a) Schematic of the one-dimensional trimer model. b) Energy eigenvalues as a function of the inter-cell coupling $t_{3}$ under the inversion-symmetric condition. c) Energy spectrum with a parameter ratio of $t_{3}/t_{1}=5$. d) Amplitude distribution of the topological edge state located in the bandgap of (c).

In this work, we construct the domain wall using trimer lattices in distinct topological phases. The right half of the structure is formed by truncating the topological lattice. This truncation is designed to eliminate the topological edge mode at the rightmost boundary. Specifically, the two rightmost lattice sites of the chain are removed. The energy spectrum of this truncated lattice is shown in Figure S2a. Compared to the complete lattice spectrum in Figure S1c, the change of boundary symmetry results in only two topological edge states surviving in the band gap. The eigenstate amplitude distribution is shown in Figure S2b. The topological mode now exists on the left boundary of the lattice.

**Figure S2.** Topological mode in the truncated trimer lattice. a) Energy spectrum of the topological lattice after removing the two rightmost lattice sites. The calculation parameters are identical to those in Figure S1c. b) Amplitude distribution of the topological edge state, demonstrating its localization strictly at the left boundary.

The left half of the domain wall structure employs a parameter configuration that breaks the inversion symmetry. Previous studies indicate that certain parameter configurations allow the existence of edge states even in the absence of inversion symmetry.^[1]^ To obtain a purely trivial structure completely devoid of any edge states, the parameters are specifically set to $t_{1}=t_{3}<t_{2}$. Figure S3 shows the calculated energy spectrum of the lattice under this symmetry-broken configuration, with a specific parameter ratio of $t_{1}/t_{2}=1/5$. It is observed that a clear band gap exists between the energy bands, and no topological states appear within the gap. This specific lattice lacking edge modes constitutes the left part of the domain wall system. The complete domain wall structure presented in this work is constructed by spatially connecting the trivial lattice on the left and the truncated topological lattice on the right. This design ensures overall structural symmetry while realizing the stable interface states.

**Figure S3.** Energy spectrum of the trivial trimer lattice. Energy eigenvalues for a lattice with broken inversion symmetry. The bandgap is completely devoid of any localized edge modes.

**S2. Comparison of the interface transmission spectra**

Figure S4 overlays the measured transmission spectra at the two interface waveguides, corresponding to Figures 2b and 2c in the main text. The two curves exhibit out-of-phase oscillations as the wavelength is varied, consistent with energy exchange between the interface waveguides. The wavelength dependence of the coupling coefficients changes the spatial beating period, producing complementary variations in the output powers at the fixed device length.

**Figure S4**. Comparison of the measured transmission spectra at the two interface waveguides. The spectra at sites −1 and +1, reproduced from Figures 2b and 2c in the main text, are plotted together to show their out-of-phase oscillations with wavelength.

**S3. Theoretical Analysis of Four-Wave Mixing in the Domain Wall**

The spatial evolution equations for four-wave mixing (FWM) in a single waveguide can be expressed as:

$$\begin{aligned} \frac{dA_{p}}{dz}=i\gamma\left( \left| A_{p} \right|^{2}+2\left| A_{s} \right|^{2}+2\left| A_{i} \right|^{2} \right)A_{p}+2i\gamma A_{p}^{*}A_{s}A_{i}e^{i\Delta\beta z}\#\left( S3 \right) \end{aligned}$$

$$\begin{aligned} \frac{dA_{s}}{dz}=i\gamma\left( \left| A_{s} \right|^{2}+2\left| A_{p} \right|^{2}+2\left| A_{i} \right|^{2} \right)A_{s}+i\gamma A_{p}^{2}A_{i}^{*}e^{-i\Delta\beta z}\#\left( S4 \right) \end{aligned}$$

$$\begin{aligned} \frac{dA_{i}}{dz}=i\gamma\left( \left| A_{i} \right|^{2}+2\left| A_{p} \right|^{2}+2\left| A_{s} \right|^{2} \right)A_{i}+i\gamma A_{p}^{2}A_{s}^{*}e^{-i\Delta\beta z}\#\left( S5 \right) \end{aligned}$$

When implementing FWM in a waveguide array, we apply the undepleted pump approximation and neglect the self-phase modulation (SPM) and cross-phase modulation (XPM) of the signal and idler. In this work, the utilized pump pulses feature relatively large pulse widths and low peak powers. Consequently, the Kerr nonlinear effects induced by the pump are not prominent and do not significantly influence the spatial coupling dynamics [2]. The coupled-mode equations therefore only retain the FWM terms for the signal and idler. The spatial evolution equations for the pump, signal, and idler in the *n*-th waveguide are given by:

$$\begin{aligned} \frac{dA_{p,n}}{dz}=i\kappa_{p,n-1}A_{p,n-1}+{i\kappa_{p,n}A}_{p,n+1}\#\left( S6 \right) \end{aligned}$$

$$\begin{aligned} \frac{dA_{s,n}}{dz}=i\kappa_{s,n-1}A_{s,n-1}+i\kappa_{s,n}A_{s,n+1}+i\gamma A_{p,n}^{2}\left( z \right)A_{i,n}^{*}\left( z \right)e^{-i\Delta\beta z}\#\left( S7 \right) \end{aligned}$$

$$\begin{aligned} \frac{dA_{i,n}}{dz}=i\kappa_{i,n-1}A_{i,n-1}+i\kappa_{i,n}A_{i,n+1}+i\gamma A_{p,n}^{2}\left( z \right)A_{s,n}^{*}\left( z \right)e^{-i\Delta\beta z}\#\left( S8 \right) \end{aligned}$$

Where $\kappa_{p,n}$，$\kappa_{s,n}$，$\kappa_{i,n}$ denote the linear coupling coefficients for the pump, signal, and idler between the *n*-th and (*n*+1)-th waveguides, respectively. The boundary conditions of the array are defined as:

$$\begin{aligned} \kappa_{p,0}=\kappa_{s,0}=\kappa_{i,0}=0\#\left( S9 \right) \end{aligned}$$

$$\begin{aligned} \kappa_{p,N}=\kappa_{s,N}=\kappa_{i,N}=0\#\left( S10 \right) \end{aligned}$$

According to our theoretical analysis of the lattice and linear optical experimental results, light injected into the domain wall of the trimer lattice is strongly localized within the two interface waveguides, exhibiting a beating behavior during propagation. To simplify the model, we restrict our analysis to the light evolution within these two waveguides at the domain wall. We denote these two waveguides as index -1 and +1. The evolution equations are simplified to:

$$\begin{aligned} \frac{dA_{p,-1}}{dz}=i\kappa_{p}A_{p,+1}\#\left( S11 \right) \end{aligned}$$

$$\begin{aligned} \frac{dA_{p,+1}}{dz}=i\kappa_{p}A_{p,-1}\#\left( S12 \right) \end{aligned}$$

$$\begin{aligned} \frac{dA_{s,-1}}{dz}=i\kappa_{s}A_{s,+1}+i\gamma A_{p,-1}^{2}\left( z \right)A_{i,-1}^{*}\left( z \right)e^{-i\Delta\beta z}\#\left( S13 \right) \end{aligned}$$

$$\begin{aligned} \frac{dA_{s,+1}}{dz}=i\kappa_{s}A_{s,-1}+i\gamma A_{p,+1}^{2}\left( z \right)A_{i,+1}^{*}\left( z \right)e^{-i\Delta\beta z}\#\left( S14 \right) \end{aligned}$$

$$\begin{aligned} \frac{dA_{i,-1}}{dz}=i\kappa_{i}A_{i,+1}+i\gamma A_{p,-1}^{2}\left( z \right)A_{s,-1}^{*}\left( z \right)e^{-i\Delta\beta z}\#\left( S15 \right) \end{aligned}$$

$$\begin{aligned} \frac{dA_{i,+1}}{dz}=i\kappa_{i}A_{i,-1}+i\gamma A_{p,+1}^{2}\left( z \right)A_{s,+1}^{*}\left( z \right)e^{-i\Delta\beta z}\#\left( S16 \right) \end{aligned}$$

To analytically understand the influence of waveguide coupling on the idler conversion efficiency, we first consider the weak-gain limit. In this case, the signal amplitude is assumed to be unaffected by the nonlinear gain, and its behavior is dominated by the linear spatial coupling. The evolution equations for the signal thus take a similar form to those of the pump:

$$\begin{aligned} \frac{dA_{s,-1}}{dz}=i\kappa_{s}A_{s,+1}\#\left( S17 \right) \end{aligned}$$

$$\begin{aligned} \frac{dA_{s,+1}}{dz}=i\kappa_{s}A_{s,-1}\#\left( S18 \right) \end{aligned}$$

Assuming both the pump and signal are incident exclusively from waveguide -1, we obtain the evolution solutions:

$$\begin{aligned} A_{p,-1}\left( z \right)=A_{p0}\cos\left( \kappa_{p}z \right)\#\left( S19 \right) \end{aligned}$$

$$\begin{aligned} A_{p,+1}\left( z \right)={iA}_{p0}\sin\left( \kappa_{p}z \right)\#\left( S20 \right) \end{aligned}$$

$$\begin{aligned} A_{s,-1}\left( z \right)=A_{s0}\cos\left( \kappa_{s}z \right)\#\left( S21 \right) \end{aligned}$$

$$\begin{aligned} A_{s,+1}\left( z \right)={iA}_{s0}\sin\left( \kappa_{s}z \right)\#\left( S22 \right) \end{aligned}$$

Substituting these spatial profiles into the idler equations yields two nonlinear driving terms for the respective waveguides:

$$\begin{aligned} {f_{-1}\left( z \right)=i\gamma A_{p,-1}^{2}\left( z \right)A_{s,-1}^{*}\left( z \right)e^{-i\Delta\beta z}=i\gamma A_{p0}^{2}A_{s0}^{*}\cos}^{2}\left( \kappa_{p}z \right)\cos\left( \kappa_{s}z \right)e^{-i\Delta\beta z}\#\left( S23 \right) \end{aligned}$$

$$\begin{aligned} {f_{+1}\left( z \right)=i\gamma A_{p,+1}^{2}\left( z \right)A_{s,+1}^{*}\left( z \right)e^{-i\Delta\beta z}=-\gamma A_{p0}^{2}A_{s0}^{*}\sin}^{2}\left( \kappa_{p}z \right)\sin\left( \kappa_{s}z \right)e^{-i\Delta\beta z}\#\left( S24 \right) \end{aligned}$$

The idler evolution equations are thereby rewritten as:

$$\begin{aligned} \frac{dA_{i,-1}}{dz}=i\kappa_{i}A_{i,+1}+f_{-1}\left( z \right)\#\left( S25 \right) \end{aligned}$$

$$\begin{aligned} \frac{dA_{i,+1}}{dz}=i\kappa_{i}A_{i,-1}+f_{+1}\left( z \right)\#\left( S26 \right) \end{aligned}$$

Solving this coupled system yields the idler amplitude at distance *L*:

$$\begin{aligned} \begin{aligned} A_{i,-1}\left( L \right)=\int_{0}^{L} \left[ \cos\left( t_{i}\left( L-z \right) \right)f_{-1}\left( z \right)+i\sin\left( t_{i}\left( L-z \right) \right)f_{+1}\left( z \right) \right]dz \end{aligned}\#\left( S27 \right) \end{aligned}$$

We evaluate this integral at a propagation length *L* where the idler completely couples back to waveguide -1. We further define the coupling coefficient dispersion as $\kappa_{s}-\kappa_{p}=\kappa_{p}-\kappa_{i}=\delta$. Under ideal phase-matching conditions ($\Delta\beta=0$) and after omitting the rapidly oscillating terms, the integral calculation simplifies to the analytical amplitude of the idler in waveguide -1:

$$\begin{aligned} A_{i,-1}\left( L \right)=i\gamma A_{p0}^{2}A_{s0}^{*}\frac{L}{4}\#\left( S28 \right) \end{aligned}$$

As a comparison, spatial oscillation is absent in a single decoupled waveguide, leading to a linear accumulation of the idler amplitude:

$$\begin{aligned} A_{i,single}\left( L \right)=i\gamma A_{p0}^{2}A_{s0}^{*}L\#\left( S29 \right) \end{aligned}$$

The corresponding idler intensities $I=\left| A \right|^{2}$ for the two-waveguide system and the single waveguide are given by:

$$\begin{aligned} I_{i,-1}\left( L \right)=\left| A_{i,-1}\left( L \right) \right|^{2}=\left| \gamma A_{p0}^{2}A_{s0}^{*} \right|^{2}\frac{L^{2}}{16}\#\left( S30 \right) \end{aligned}$$

$$\begin{aligned} I_{i,single}\left( L \right)=\left| A_{i,single}\left( L \right) \right|^{2}=\left| \gamma A_{p0}^{2}A_{s0}^{*} \right|^{2}L^{2}\#\left( S31 \right) \end{aligned}$$

This indicates that the idler intensity in the coupled two-waveguide system is 1/16 of that in a single waveguide case.

We further investigate a specific condition where all coupling coefficients are uniform ($\kappa_{p}=\kappa_{s}=\kappa_{i}$). Integrating the equations under this assumption yields:

$$\begin{aligned} A_{i,-1}\left( L \right)=i\gamma A_{p0}^{2}A_{s0}^{*}\frac{3L}{4}\#\left( S32 \right) \end{aligned}$$

The corresponding idler intensity under this uniform coupling condition is:

$$\begin{aligned} I_{i,-1}\left( L \right)=\left| A_{i,-1}\left( L \right) \right|^{2}=\left| \gamma A_{p0}^{2}A_{s0}^{*} \right|^{2}\frac{9L^{2}}{16}\#\left( S33 \right) \end{aligned}$$

In this case, the theoretical idler intensity in the two-waveguide system is 9/16 of the single waveguide intensity. This analytical derivation explains the characteristic intensity difference observed in Figure 4b.

However, when the nonlinear driving parameter (defined as $g=\gamma A_{p0}^{2}$) is significantly larger, the signal intensity also experiences prominent parametric amplification. Under such conditions, the idler grows exponentially with the propagation distance, and the conversion efficiency decrease induced by spatial coupling becomes greater. As illustrated in Figure S5, the difference in idler intensity between the trimer lattice domain wall and the single waveguide grows larger as the nonlinear driving parameter *g* increases.

**Figure S5.** Idler intensity evolution along the propagation distance for a single waveguide (red dashed line) and the trimer lattice domain wall (blue solid line) under different nonlinear driving parameters: (a) *g* = 200, (b) *g* = 500, and (c) *g* = 1000.

**S4. Fabrication of the topological waveguide arrays**

The topological waveguides are fabricated on the USRN platform. Initially, a 300-nm-thick USRN film is deposited onto a silicon substrate covered by a 3-μm-thick thermal SiO2 layer. This deposition step is executed via inductively coupled plasma chemical vapor deposition at a low temperature of 250 °C. Subsequently, an electron-beam resist is spin-coated onto the sample, and the waveguide patterns are defined utilizing electron-beam lithography. Inductively coupled plasma etching is then applied to transfer the structures into the USRN layer, which precisely constructs the domain wall interface of the device. Following the removal of the remaining resist, a 2-μm-thick SiO2 upper cladding is grown using a combination of atomic layer deposition and plasma-enhanced chemical vapor deposition. In terms of optical properties, USRN features linear and nonlinear refractive indices of 3.1 and $2.8\times{10}^{-13}cm^{2}W^{-1}$, respectively. Given its bandgap of 2.1 eV, the two-photon absorption edge of USRN is situated at 1.2 μm, which completely precludes two-photon absorption at our experimental wavelengths.

**S5. Calculation of Signal Gain and Idler Conversion Efficiency**

We evaluate the signal gain and idler conversion efficiency from the measured optical spectra. The integration covers the full signal band, including the sidebands. Background contributions are subtracted before integration. Given the pulsed excitation, the peak power of the signal is extracted as:

$$\begin{aligned} P_{signal, peak}=\frac{1}{R_{p}T}\int P_{signal,ave}\left( \lambda\right)d\lambda\#\left( S34 \right) \end{aligned}$$

Here, $P_{\text{signal,}\text{ }\text{ave}}\left( \lambda\right)$ is the measured average output signal spectrum after subtraction of the signal-only and pump-only background contributions. $R_{p}$ is the pulse repetition rate (20 MHz) and $T$ is the pulse width (5 ps). The factor $R_{p}T$ accounts for the pump duty cycle. Similarly, the peak power of the generated idler is calculated from its average spectrum $P_{\text{idler,}\text{ }\text{ave}}\left( \lambda\right)$:

$$\begin{aligned} P_{idler, peak}=\frac{1}{R_{p}T}\int P_{idler,ave}\left( \lambda\right)d\lambda\#\left( S35 \right) \end{aligned}$$

The signal gain is defined as $G_{\text{signal}}=P_{\text{signal,}\text{ }\text{peak}}/P_{\text{signal,}\text{ }\text{out}}$, where $P_{\text{signal,}\text{ }\text{out}}$ is the transmitted signal power with the pump turned off. The idler conversion efficiency is calculated as the ratio of the generated idler peak power to this reference signal power, $C.E._{\text{idler}}=P_{\text{idler,}\text{ }\text{peak}}/P_{\text{signal,}\text{ }\text{out}}$.

**SI References**

1.	Martinez Alvarez, V. M. & Coutinho-Filho, M. D. Edge states in trimer lattices. *Phys. Rev. A* 99, 013833 (2019).

2.	Sohn, B.-U. et al. A topological nonlinear parametric amplifier. *Nat. Commun.* 13, 7218 (2022).
